# Supplementary material for: HIVprotI: an integrated web based platform for prediction and design of HIV proteins inhibitors
Source: J Cheminform. 2018 Mar 9;10:12. doi: 10.1186/s13321-018-0266-y (PMC5845081; doi:10.1186/s13321-018-0266-y)
Supplement: Supplementary file 2 — Additional file 2. Source code of HIVProtI web server. [file 13321_2018_266_MOESM2_ESM.zip › HIVprotI_Source-code/hivprotI/weka-3-6-6/doc/weka/gui/streams/package-summary.html]

weka.gui.streams


---


|  |  |  |
| --- | --- | --- |
| **Overview** \| **Package** \| Class \| **Tree** \| **Deprecated** \| **Index** \| **Help** \| **Weka's home** | |  |
| **PREV PACKAGE**   **NEXT PACKAGE** | **FRAMES**    **NO FRAMES**     **All Classes** |  |


---

## Package weka.gui.streams

| **Interface Summary** | |
| --- | --- |
| **InstanceListener** | An interface for objects interested in listening to streams of instances. |
| **InstanceProducer** | An interface for objects capable of producing streams of instances. |
| **SerialInstanceListener** | Defines an interface for objects able to produce two output streams of instances. |

| **Class Summary** | |
| --- | --- |
| **InstanceCounter** | A bean that counts instances streamed to it. |
| **InstanceEvent** | An event encapsulating an instance stream event. |
| **InstanceJoiner** | A bean that joins two streams of instances into one. |
| **InstanceLoader** | A bean that produces a stream of instances from a file. |
| **InstanceSavePanel** | A bean that saves a stream of instances to a file. |
| **InstanceTable** | A bean that takes a stream of instances and displays in a table. |
| **InstanceViewer** | This is a very simple instance viewer - just displays the dataset as text output as it would be written to a file. |
| **Messages** | Messages. |

---


|  |  |  |
| --- | --- | --- |
| **Overview** \| **Package** \| Class \| **Tree** \| **Deprecated** \| **Index** \| **Help** \| **Weka's home** | |  |
| **PREV PACKAGE**   **NEXT PACKAGE** | **FRAMES**    **NO FRAMES**     **All Classes** |  |


---
